# Are Human Digit Muscles Devoid of Recurrent Inhibition?

**DOI:** 10.3389/fncel.2015.00507

**Published:** 2016-01-11

**Authors:** Maria Piotrkiewicz, Dariusz Młoźniak

**Affiliations:** Engineering of Nervous and Muscular System, Nałęcz Institute of Biocybernetics and Biomedical Engineering, Polish Academy of SciencesWarsaw, Poland

**Keywords:** recurrent inhibition, afferents, Ia non-reciprocal inhibition, Ib interneuron, motoneurons, digit muscles, human

Recurrent inhibition (RI), mediated by Renshaw cells (Renshaw, 1941; Eccles et al., 1954) have been extensively studied in animals for over 70 years (reviewed in Windhorst, 1996; Alvarez and Fyffe, 2007). It attracts considerable interest to this day, since its role in motor control still remains to be elucidated.

Several studies have examined the distribution of RI in motoneuron (MN) pools supplying various cat and human muscles. Illert and colleagues found recurrent axon collaterals in all explored motor nuclei except those of the digit muscles (Illert and Wietelmann, 1989; Höerner et al., 1991); see also the review by Illert and Kümmel (1999). In man the RI distribution in various motor nuclei was studied mostly by paired H reflex technique (Pierrot-Deseilligny and Bussel, 1975; Bussel and Pierrot-Deseilligny, 1977), which is based on the analysis of surface EMG after supramaximal nerve stimulation. The results were essentially consistent with animal studies mentioned above (Katz and Pierrot-Deseilligny, 1999). Also in the single motor unit (MU) study Person and Kozhina (1978) did not find RI in abductor pollicis and abductor digiti minimi. Although other researches provided evidence for weak RI in cat digit muscles (Thomas and Wilson, 1967; Hamm, 1990; McCurdy and Hamm, 1992; Turkin et al., 1998), it is commonly believed that RI in MN pools supplying digit muscles does not exist.

Presumably that is why Inglis et al. (1997) did not take RI into account as a possible explanation of the clear inhibition (their Figure 2B) observed in a MN supplying the human long flexor of the thumb. More evidence for short-latency inhibition in abductor digiti minimi and first dorsal interosseus was presented recently during 7th International MN Meeting in Paris (Piotrkiewicz et al., 2010). The interpretation of this inhibition (based on its latency) as the evidence for RI was met with strong disbelief.

In view of these conflicting opinions, the question arose whether the short-latency inhibition observed in human digit muscles could indeed be evoked by Renshaw cells, or is there any alternative explanation.

There are multiple synaptic influences converging on a MN. The spinal pathways, which could be responsible for short-latency inhibition, are schematically presented in Figure 1A. They include RI and two other pathways, both mediated by the Ib inhibitory interneurons. The latter pathways were investigated in cat by Jankowska and colleagues (Brink et al., 1983; Harrison and Jankowska, 1985; Jankowska and Zytnicki, 1985). Special attention was paid to the so-called non-reciprocal Ia inhibition (Ia NRI), which was observed after excitation of Ia afferents with very weak stimulus. The presence of Ia NRI was documented in several muscles, including flexor digitorum longus, hallucis longus, and intrinsic foot muscles. Ib inhibitory interneurons could also be excited in the same experiment by stimulation of Ib afferents, which however required considerably stronger stimuli. Al three types of inhibition are disynaptic, so their latencies are very close to each other. Therefore, it would be impossible to distinguish RI from the inhibition mediated by Ib interneurons solely on the basis of their latencies.

**Figure 1:**
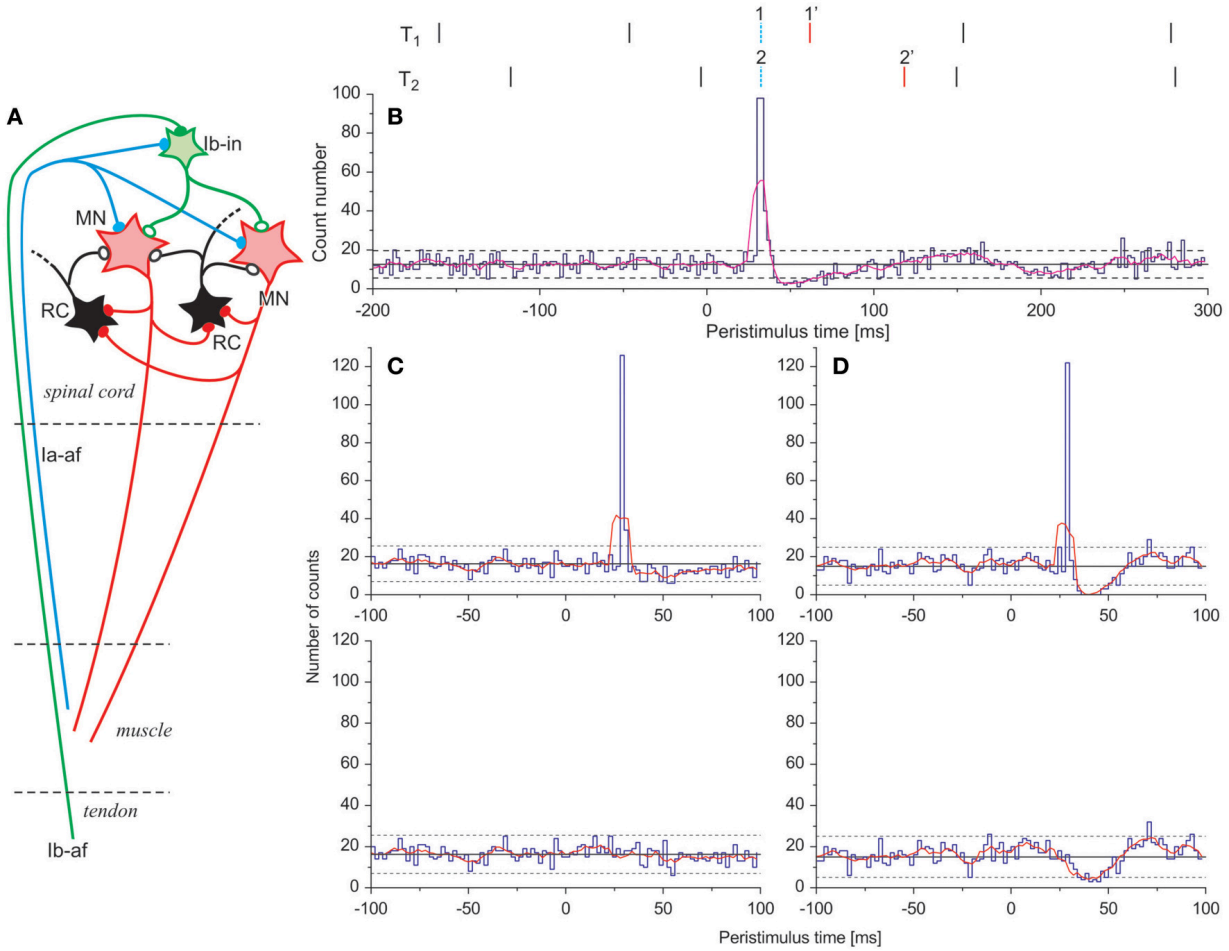
**Short-latency inhibition**. **(A)** Scheme of spinal circuits: MN, motoneurons; RC, Renshaw cells; Ia-af, Ia afferent; Ib-aff, Ib afferent; Ib-in, Ib interneuron; heavy buttons, excitatory synapses, open buttons, inhibitory synapses. **(B)** Principle of “gap filling.” Bottom, example of PSTH from computer simulations (single EPSP with rise time of 3.8 ms and amplitude of 1.5 mV). Red line, moving average calculated from 5 consecutive PSTH values. Top, schematic explanation of “gap filling” procedure: T1, T2, trains of MN discharges time-locked with two separate stimuli (vertical bars); discharges 1 and 2, synchronized in the excitation peak (dotted bars), are moved to the positions 1′ and 2′. **(C)** and **(D)** Results of “gap filling” procedure applied to simulated PSTHs (upper graph, original data, lower graph, data after “gap filling”): **(C)** single excitatory potential with rise time 1.2 ms and amplitude 1 mV; **(D)** volley composed of the same excitatory potential and inhibitory potential with rise time 16 ms and amplitude -4 mV). The Figure was adopted from Piotrkiewicz et al. (2010).

In experiments conducted by Kudina and Andreeva (Piotrkiewicz et al., 2010) the stimuli were delivered to the ulnar nerve during weak muscle contractions, evoking activity of a few single MUs. The stimulus intensity was kept slightly above threshold of the M-response. The stimulus-correlated MU discharge patterns were analyzed by means of peristimulus time histogram (PSTH, see Figure 1B). In virtually all single responses PSTHs contained a short-latency excitatory peak. The latencies (25–30 ms) indicated that this excitation was mediated by Ia afferents.

Any excitatory peak is followed by a period of depression of the duration approximately equal to the mean interspike interval (Figure 1B). The possible short-latency inhibition would arrive to the MN a few ms later, so it would merge with this depression and may not be recognized. That is why the PSTHs containing excitation peaks were not normally analyzed with respect to possible inhibition, although Binboğa et al. (2011) hypothesized that certain patterns of the reflex responses observed on the frequencygrams in their experimental study might be attributed to the excitation-inhibition volley.

To overcome this difficulty, we developed the method that we called “gap filling.” It is explained here on the examples from computer simulations based on the simple threshold-crossing model described elsewhere (Piotrkiewicz, 1999; Piotrkiewicz and Kudina, 2012). The model was verified by comparison with results of several human experiments (Kuraszkiewicz et al., 2012).

The method allows extinguishing both H-reflex peak and following depression. This makes possible the clear distinction between cases where inhibition is present and where it is not, as well as the quantitative evaluation of the inhibition, when it is present. The essence of the method consists of shifting discharges synchronized in the excitatory peak by substitution of each interval from the previous discharge by the one chosen from the distribution of background intervals (see upper panel of Figure 1B). If the stimulus-induced synaptic volley is due to the single Ia EPSP, “gap filling” causes the disappearance of the peak and filling up the following depression trough (Figure 1C). If Ia EPSP is followed by an IPSP, after application of “gap filling” the impact of IPSP is clearly visualized (Figure 1D).

Since in the all described above experiments in human digit muscles Ia excitation was present, the observed inhibition could be due to Ia NRI. The stimuli were comparatively weak, which according to Brink et al. (1983) made the excitation of Ib afferents unlikely. On the other hand, the stimulation was strong enough to evoke the motor response, and it has been shown that single Ib afferents can be excited by the twitch of single MU (Binder et al., 1977). In human experiments it is rather difficult to estimate how many MUs contributed to M-response and whether Ib afferents which were excited, could evoke the noticeable inhibition in the given MN. Thus, it is impossible to decide if Ib afferents could contribute to the inhibition observed.

Inglis et al. (1997) suggested that their example of short-latency inhibition presented in flexor pollicis longus could be due to Ib inhibition. The possibility that this inhibition could be caused by Ia NRI is very unlikely, since the study revealed that homonymous Ia projections were very weak in this muscle. However, in their experiments stimulation was rather weak (only 10% higher than the motor threshold) and the presence of M-response, which is the sine-qua-non condition for evoking RI, suggests that this possibility should also be taken into account.

The lack of evidence cannot be interpreted as the evidence of non-existence. Thus, the evidence of RI in some of cat muscles proves that there is no reason to believe that human digit muscles are devoid of RI. It should be, however, stressed that in experiments on human digit muscles RI was observed in only 46% of MNs (13 from 28). These results combined with results from animal studies indicate the existence of proximo-distal gradient in RI intensity.

Summing up, the question: “What is the real nature of the described short-latency inhibition in human digit muscles?” will remain unresolved. Both RI and inhibition mediated by Ib interneurons are possible.

As it was discussed above, short-latency inhibition of one or another origin does exist in MN pools controlling digit muscles, despite some opinions trying to justify RI absence. This indicates the importance of inhibitory circuits in motor control. It was recently shown in the modeling study (Friedel and Van Hemmen, 2008) that inhibition is essential for high-quality multimodal sensory integration. This makes the search for the role of the short-latency inhibition even more exciting. To the best of our knowledge, the Ia NRI was not studied in single human MUs. Thus, new experiments are necessary to elucidate the possible contributions of existing inhibition mechanisms to the control of MNs supplying human digit muscles. Our method for studying short-latency inhibition in the presence of Ia excitation can reveal the possible presence of RI in ongoing experiments as well as be applied to existing data.

## Author contributions

MP proposed the subject and prepared article draft. DM developed the software for EMG decomposition and analyzed the experimental data, on which the opinion was based. Both authors discussed the text and contributed to its final shape.

### Conflict of interest statement

The authors declare that the research was conducted in the absence of any commercial or financial relationships that could be construed as a potential conflict of interest.
